# Tandem LTR-retrotransposon structures are common and highly polymorphic in plant genomes

**DOI:** 10.1186/s13100-025-00347-y

**Published:** 2025-03-12

**Authors:** Noemia Morales-Díaz, Svitlana Sushko, Lucía Campos-Dominguez, Venkataramana Kopalli, Agnieszka A. Golicz, Raúl Castanera, Josep M. Casacuberta

**Affiliations:** 1 Centre for Research in Agricultural Genomics, CRAG (CSIC- IRTA-UAB-UB), Campus UAB, Cerdanyola del Vallès, Barcelona, Spain; 2 Department of Plant Breeding, Justus Liebig University Giessen, Giessen, Germany; 3 IRTA, Genomics and Biotechnology, Edifici CRAG, Campus UAB, Bellaterra, Catalonia 08193 Spain; 4 Present Address: Department of Molecular Biology, Max Planck Institute for Biology Tübingen, Tübingen, Germany

## Abstract

**Background:**

LTR-retrotransposons (LTR-RT) are a major component of plant genomes and important drivers of genome evolution. Most LTR-RT copies in plant genomes are defective elements found as truncated copies, nested insertions or as part of more complex structures. The recent availability of highly contiguous plant genome assemblies based on long-read sequences now allows to perform detailed characterization of these complex structures and to evaluate their importance for plant genome evolution.

**Results:**

The detailed analysis of two rice loci containing complex LTR-RT structures showed that they consist of tandem arrays of LTR copies sharing internal LTRs. Our analyses suggests that these LTR-RT tandems are the result of a single insertion and not of the recombination of two independent LTR-RT elements. Our results also suggest that gypsy elements may be more prone to form these structures. We show that these structures are highly polymorphic in rice and therefore have the potential to generate genetic variability. We have developed a computational pipeline (IDENTAM) that scans genome sequences and identifies tandem LTR-RT candidates. Using this tool, we have detected 266 tandems in a pangenome built from the genomes of 76 accessions of cultivated and wild rice, showing that tandem LTR-RT structures are frequent and highly polymorphic in rice. Running IDENTAM in the Arabidopsis, almond and cotton genomes showed that LTR-RT tandems are frequent in plant genomes of different size, complexity and ploidy level. The complexity of differentiating intra-element variations at the nucleotide level among haplotypes is very high, and we found that graph-based pangenomic methodologies are appropriate to resolve these structures.

**Conclusions:**

Our results show that LTR-RT elements can form tandem arrays. These structures are relatively abundant and highly polymorphic in rice and are widespread in the plant kingdom. Future studies will contribute to understanding how these structures originate and whether the variability that they generate has a functional impact.

**Supplementary Information:**

The online version contains supplementary material available at 10.1186/s13100-025-00347-y.

## Background

Transposable Elements (TEs) are a major component of eukaryote genomes. In plants, TEs frequently account for most of the genome content, as for example in maize where TE-related sequences account for 85% of the genome content [1]. TEs contribute to genome evolution in many ways, fulfilling structural roles and generating genome variability that can translate into phenotypic novelty [2]. In plants, LTR retrotransposons (LTR-RTs), together with MITEs, are the most prevalent types of TEs [3]. As an example, in maize LTR-RTs account for as much as 90% of the total TE content [1]. LTR-RT insertions can inactivate genes or result in changes of the expression of genes located nearby and be at the origin of new phenotypic variability [3, 4]. In fact, different LTR-RT insertions have been selected during the domestication, local adaptation and breeding of plant crops [5]. LTR-RTs move through a replicative process, which leads to increasing their copy number while transposing. Their amplification can be at the origin of rapid increases in genome size, as has been shown in *O. australiensis*, where the genome size doubling in just three million years can be explained by the amplification of three families of LTR-RTs [6]. However, LTR-RT sequences can also be eliminated from genomes, thus reverting the tendency to genome size expansion [7, 8]. The main mechanism for this is illegitimate recombination, either at the LTRs giving rise to the so called solo-LTRs or involving any other repeated sequence thus resulting in truncated LTR-copies [9–12]. In fact, most LTR-RT-related sequences in genomes are deletion derivatives of LTR-RTs and are no longer able to autonomously transpose [13]. Moreover, LTR-RTs can also give rise to complex LTR-RT-related structures through their nested insertion in other LTR-RTs. For example, the array of nested LTR-RTs of different families is common in the genomes of maize and barley [11, 14]. This can be explained by the lack of phenotypic consequences of the insertion in these gene-free regions, and therefore the lower selective pressure against these insertions, or by a targeted insertion of certain LTR-RT families. For example, the latter could be the case of the two main LTR-RT families of *Physcomitrium patens*, RLG1 and RLC5 that are generally found forming heterochromatic islands composed mainly of a single family LTR-RT elements in the chromosome arms and the centromere, respectively [15–17]. In addition, a particular type of defective LTR-RT, called Terminal-repeat Retrotransposons in Miniature, TRIMs, has been shown to form tandem repeats of elements sharing an internal LTR [18, 19]. These structures can be the result of illegitimate recombination or could be generated during the retrotransposition process [18, 19]. Tandem repeats of LTR-RT sequences sharing internal LTRs have also been found in the centromeres of two species of kangaroos [20], and it has been proposed that they could be originated by illegitimate recombination when repairing a double-strand break (DSB) at an LTR with the other LTR of the element from the sister chromatid or the homologous chromosome [21]. Moreover, it has been reported in yeast that LTR-RTs could also generate these tandem structures through an integrase-independent mechanism of integration into preexisting elements [22, 23].

Previous data on Drosophila suggests that tandem TE insertions may be relatively frequent in eukaryote genomes [24]. Although the available plant reference genomes probably contain TE tandem repeats, these have not been systematically analyzed or reported due to the difficulty to discard the artefactual nature of some of these structures when the reference genomes are based mainly on short read data. However, as the number of Telomere-to-Telomere and high-quality assemblies based on long-read data increases, it becomes feasible to analyze the structure and the prevalence of tandem-repeat LTR-RT insertions in plant genomes. Here we show that plant genomes frequently have tandem arrays of LTR-RTs sharing the internal LTRs and that these structures are highly variable, thus increasing the potential of LTR-RTs for generating phenotypic variability.

## Methods

### Detecting LTR-RT tandems and intact LTR-RTs

To detect LTR-RT tandems in different plant genomes, we developed a bioinformatics pipeline, IDENTAM (https://github.com/NMoralesD/IDENTAM), which requires a defined LTR-RT consensus library with internal and LTR regions provided as separate sequences and a reference genome. This pipeline uses a RepeatMasker output to identify the hits that cover more than 70% of the consensus length and employs two approaches (modules) to detect tandems. Module 1 identifies two nearby LTR-RT internal regions, and module 2 detects three close LTRs. Multiple filters (flexible parameters set by the user) can be applied to limit false positives, and TEsorter [25] is then applied to classify the elements in two categories: (i) LTR-RT_TR, which are potential LTR-RT tandems with recognized coding domains and associated to known LTR-RT lineages, or (ii) LTR-RT-related, which are tandemly arranged elements without recognized coding domains. The pipeline parameters used in this study were: maximum distance between internal regions of 5.000 bp for module 1, and maximum distance between LTRs of 15.000 bp for module 2. For both modules, the minimum LTR-TR internal region size was set to 500 bp, the minimum element size to 1000 bp, and the maximum locus size to 40,000 bp. An expanded description of this pipeline is shown in Additional Fig. 1. Input LTR-RT libraries for IDENTAM were built with EDTA_raw.pl script [26], except for rice, in which a previously published TE library was used [26].

### Rice pangenome construction

We followed two different strategies to build a pangenome of rice. We first created a pangenome using long-read-based genome assemblies of 76 rice varieties, representing the diversity of the species and including also 7 assemblies of the wild rice relatives *O. rufipogon* (3), *O. barthii* (3) and 1 *O. glaberrima* (1) (Additional Table 1). We anchored the pangenome to the Nipponbare IRGSP-1.0 genome [27]. Every assembly was aligned to IRGSP-1.0 using minimap2 [28] and SVIM-asm [29] was used for structural variant detection. The vcf files generated by SVIM-asm for the 75 genomes were merged with bcftools merge [30] (-m none) and Truvari [31](-p 0, -P 0.5, -s 0). The pangenome graph was built using vgtools [32]. To identify the LTR-RT Tandems corresponding to transposon insertion polymorphisms (TIPs), we ran IDENTAM pipeline on the insertion and deletion sequences detected by SVIM-asm [28]. A second pangenome was obtained using Minigraph-Cactus [33] for variant detection in a reduced set of 20 accessions (Additional Table 2). The pipeline was run in every chromosome independently, and *vg deconstruct* [34] was used for variant calling using vg 1.58 version Cartari [34], with the -L parameter set to 0.9 to cluster nearly exact allele transversals. Then, large deletions (> 1 Mb) were removed using vcfbub [35] with the option -r 1,000,000. Bcftools norm with -m option [30] allowed us to split multiallelic sites into biallelic records (-). Only SVs larger than 50 bp were considered for further TE analyses. The output files were merged using *bcftools concat*, as all the files had the same columns in the same order. The pangenome graph was built again using vgtools [32].

## Results

### An LTR-RT insertion with a tandem structure in the rice genome

**Fig. 1:**
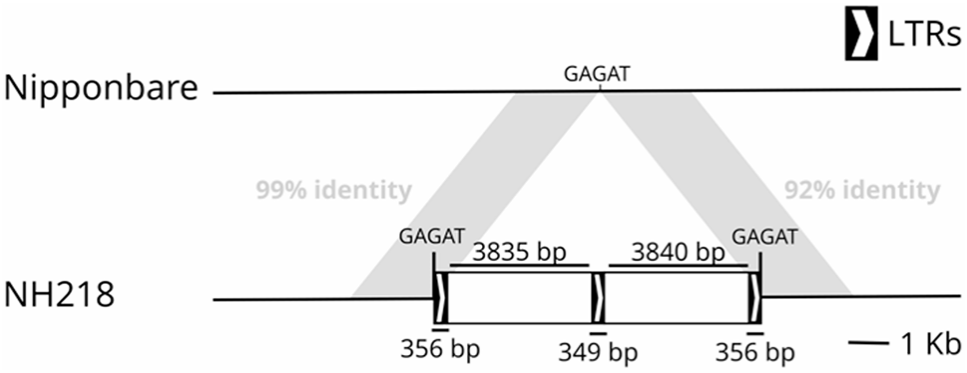
Schematic representation of the TLI1 *locus* in the Nipponbare (Chr5: 25,965,509) and NH218 genomes. The LTRs are flanked by 4 bp target site duplications (TSDs). The 2Kbp sequences flanking the tandem LTR-RT region are shown in grey

As a first step to characterize a rice non-reference LTR-RT insertion with potential phenotypic impact, as suggested by the result of a Transposon-Insertion Polymorphism GWAS (TIP-GWAS) previously performed [36], we analyzed the available long-read-based genome assemblies of different rice accessions. This analysis confirmed the presence of the LTR-RT insertion in the assembly of NH218 rice accession [37] but showed that this insertion is complex. Indeed, the insertion consists of a tandem array of two LTR-RT elements sharing an internal LTR (Fig. 1). An analysis of the long-reads used to produce the assembly of NH218 showed that the LTR-RT tandem region was covered by 4 long reads that spanned the entire LTR-RT tandem and, at least, 1 kb upstream and 5 kb downstream flanking regions. This confirmed that the tandem LTR-RT structure is not the result of an artifactual assembly and that this structure exists in the genome of the NH218 rice. We designated this insertion as Tandem LTR-RT Insertion 1 (TLI1). A comparison of the sequence with that of the Nipponbare rice reference genome (IRGSP-1.0) [27], that does not contain the insertion, shows that the insertion is accompanied by a duplication of 5 nt, which is the canonical length for the target site duplication (TSD) generated by LTR-RT upon insertion [38]. The sequences flanking the tandem LTR-RT insertion show a high degree of sequence identity (99% over 2 Kb upstream and 92% over 2 Kb downstream), which discards the tandems as being the result of the recombination of two nearby independent insertions, which would result in the elimination of the interleaving sequence. The high identity of the LTR-RT internal regions (94%) and of the LTRs (85–89%), as well as the absence of additional TSDs, also discarded the possibility of nested insertions of different LTR-RTs. Therefore, all the data suggest that the tandem LTR-RT structure is linked to a single retrotransposition-mediated insertion.

### LTR-RT tandems *loci* can be highly polymorphic

**Fig. 2:**
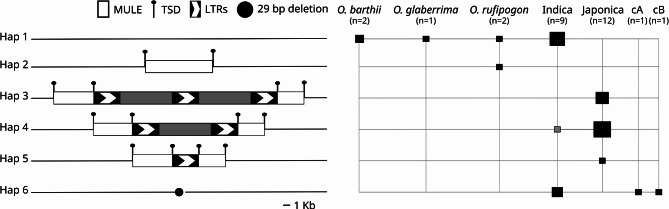
Schematic representation of the six different haplotypes observed for the TLI2 locus (left) and their relative presence in different domesticated and wild rice genome accessions (right). Black boxes indicate the presence of the haplotype in a particular species or population group, and the size of the box is proportional to the number of accessions presenting the haplotype. The grey box indicates that the haplotype Hap4 found in indica group is the result of an introgression event from japonica

The identification of a tandem LTR-RT structure in the rice genome prompted us to look more closely at other loci that appeared as complex in previous analyses. In particular, we analyzed a complex structure present in chromosome 2 of Nipponbare rice. A detailed analysis of this locus showed that it contains a tandem LTR-RT structure, with two internal regions flanked by three LTRs, inserted within a MULE transposon (Fig. 2). The MULE element is flanked by a 10 nt repeat, which fits the canonical size for MULE TSDs generated upon transposition [39] and the tandem LTR-RT is flanked by a direct repeat of 5 nt, typical for TSDs of LTR-RT insertions [38]. This suggests that the insertions are the result of two independent transposition events. As for the previous tandem LTR-RT structure analyzed, the identity of the two internal regions (99%) and the three LTRs (99%) is very high. We designated this insertion as TLI2.

An analysis of 27 additional long-read-based genome assemblies of cultivated and wild rice and related species [37, 40, 41] showed that this locus is present in at least 6 different haplotypes in these genomes (Fig. 2 and Additional Table 3). The insertion of the Mu-related element seems relatively ancient as it is found in one of the two wild rice *O. rufipogon* assemblies analyzed, although it is not present in the two assemblies of *O. barthii* and in the *O. glaberrima* assembly analyzed, which all consist of the empty site.

Interestingly, five out of the nine rice accessions belonging to the indica subspecies have the empty site (Hap 1) and all the remaining indica accessions except one (3), as well as the two aromatic japonicas analyzed have a deletion compatible with the Mu-like excision (Hap 6). We have not found any cultivated rice accession with a simple Mu-like insertion at this location, which may suggest selection for the empty site or the excision of the element. On the contrary, all the japonica accessions (12), as well as one indica accession (LARHA MUGAD, LM) contain the Mu-like insertion with a nested insertion of an LTR-RT sequence. A phylogenetic analysis of the regions flanking the insertion site (20 Kb upstream and 35 Kb downstream; Additional Fig. 2), shows that the sequences of this indica accession (LM) are more similar to those of the japonica accessions than to those of the other indica accessions, which suggest that this region may have been introgressed from japonica into the LM indica accession. Therefore, our results are compatible with the LTR-RT-related insertion happening after the split of indica and japonica and even after the split of the aromatic/circum-basmati group. We have not found any sign of excision of the Mu-like element in japonica accessions which could suggest that the nested insertion of the LTR-RT may have stabilized the Mu-like insertion. The insertion of the LTR-RT sequence consists of a tandem of two LTR-RTs sharing the internal LTR (Hap 3), a single LTR-RT insertion (Hap 4) or a solo-LTR (Hap 5).

The existence of haplotypes with single or tandem LTR-RTs and solo-LTR insertions for the same locus suggests that these structures are highly dynamic. Tandem LTR-RT insertions could be inserted as such, and single insertions (as for solo-LTRs) could be the result of illegitimate recombination event at the LTRs using the sister chromatid or the homologous chromosome. Unfortunately, the phylogenetic analysis of the sequences flanking the insertions (Additional Fig. 2) did not allow us to establish the sequence of events and discriminate between the two different mechanisms for the tandem LTR-RT formation.

### Tandems of LTR-RTs from different families are widespread in rice

To analyze how common tandem LTR-RT structures are in the rice genome, we systematically searched for these structures in the Nipponbare rice genome. To this end, we build a pipeline, that we named IDENTAM (see Methods and Additional Fig. 1), that searches for the presence of highly similar repeats of LTR-RT internal sequences interleaved with LTRs, or alternatively highly similar LTRs interleaved with LTR-RT internal sequences. We searched the Nipponbare rice reference genome [37] and identified 74 potential tandem LTR-RT structures from which 66 were clearly related to LTR-RT sequences. 89% of them are related to the *Gypsy* superfamily of LTR-RTs, and 68% of those are related to the Tekay lineage, the RIRE3 family being the most abundant among them (29%) (Additional Table 4). Although the LTR-RT tandem structure shown in Fig. 1 that prompted us to perform this analysis is found in the vicinity of an annotated gene, and some of the 66 L-RT tandems found are close to genes, these structures are more frequently found far from genes (73% are at more than 2 kb of a gene, Additional Fig. 3). A manual inspection of these 66 L-RT related insertions showed that 28 had a clear LTR-RT tandem structure (i.e. alternating internal LTR-RT sequences and LTRs, starting and finishing with an LTR) whereas the rest were potentially degenerated LTR-RT tandems, with a more complex array of LTR-RT sequences, or potential nested elements. These more complex structures were not analyzed further and were filtered out from our selection. All the selected 28 tandem LTR-RT sequences contain regions encoding conserved retrotransposon protein domains, and 10 are flanked by perfect TSD sequences of 5 nts (Additional Table 5).

**Fig. 3:**
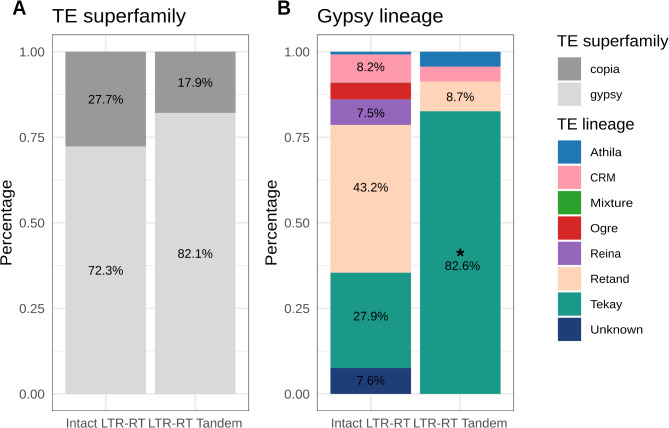
Intact LTR-RT and LTR-RT tandems in the rice Nipponbare genome. (**A**) Frequency of copia and gypsy intact LTR-RT elements and LTR-RT tandems in Nipponbare rice. (**B**) Frequency of the different gypsy lineages detected as intact LTR-RT elements and LTR-RT tandems in Nipponbare rice. The asterisk indicates statistical enrichment (*p* < 0.05) based on Fisher’s test

An analysis of the 28 L-RTs (Fig. 3) shows that most of the tandem LTR-RT insertions (82%) are related to the gypsy LTR-RT superfamily. This percentage is slightly higher than the percentage of the intact gypsy LTR-RT elements in the Nipponbare genome (72%), which could indicate that there is a slight bias in the type of elements that generate tandem LTR-RT structures. However, no significant difference was observed between the two groups (p-value = 0.2932, Fisher test). A more detailed analysis shows that 82% of the tandem LTR-RT structures related to the gypsy superfamily belong to the Tekay lineage (Fig. 3), whereas Tekay elements account only for the 28% of the gypsy elements annotated in the rice Nipponbare genome. Although the total number of the analyzed structures is low, a one-tail Fisher’s test revealed an enrichment in the Tekay linage in the LTR-RT Tandem group (p-value = 9.81e-08) which means some LTR-RT lineages are more prone to form tandem LTR-RT structures. Alternatively, the bias found could be the consequence of the particular distribution of Tekay elements, which tend to concentrate in pericentromeric regions (Additional Fig. 2). However, an analysis of the distribution of the relative distance to the centromere of the tandem LTR-RTs suggests that this may not be an important factor explaining the possible preference of Tekay elements to form tandem LTR-RT structures (Additional Fig. 4), as no specific bias is observed towards a shorter distance to the centromere. A comparison of the sequences of the LTRs as a proxy of the age of the insertions showed that 80% are more than 95% identical suggesting that they are relatively recent (Additional Fig. 5).

**Fig. 4:**
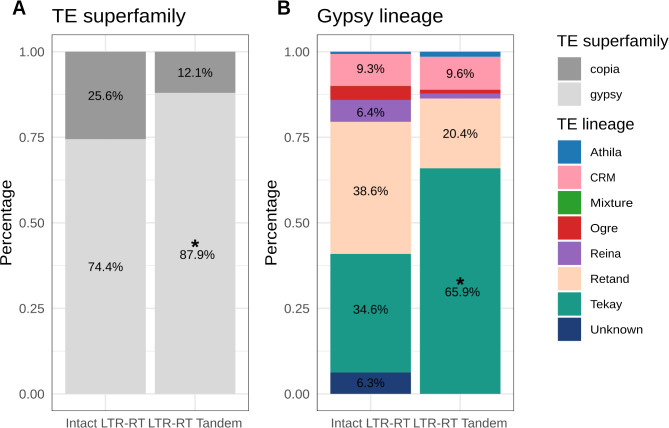
Intact LTR-RT and LTR-RT Tandems in the rice pangenome. **A**) Frequency of copia and gypsy elements identified as intact LTR-RT and LTR-RT tandem insertions in the rice pangenome. **B**) Frequency of the different gypsy lineages detected as intact LTR-RT and LTR-RT tandem insertions in the rice pangenome. The asterisk indicates statistical enrichment (*p* < 0.05) based on Fisher’s test

The analysis of the Nipponbare IRGSP-1.0 genome suggests that tandem LTR-RT structures are frequent in rice. To further analyze how frequent these structures are within rice and related species we constructed a pangenome using long-read-based genome assemblies of 75 *O. sativa* varieties, representing the diversity of the species and including also 7 assemblies of the wild rice relatives *O. rufipogon* (3), *O. barthii* (3) and *O. glaberrima* (1) [37, 41] (see methods). We found 175,555 SVs in the pangenome, which were annotated for the presence of LTR-RTs and we searched for sequences potentially corresponding to tandem LTR-RT structures using IDENTAM. We identified 200 additional tandem LTR-RT structures that are not present in the assembled genome of Nipponbare rice. On the other hand, we found that 41 out of the 66 tandem LTR-RT structures found in Nipponbare are absent from at least one of the 75 assemblies included in the pangenome. These results confirm that tandem LTR-RT structures are frequent and highly polymorphic in rice. A comparison of the types of LTR-RT elements forming the potential 266 tandem LTR-RT structures found in the pangenome (200 new non-overlapping insertions plus the 66 previously detected in Nipponbare, Fig. 4) with the LTR-RT annotation of the pangenome LTR-RTs annotated in the Nipponbare reference genome [26] plus the LTR-RT present in the SVs) shows that gypsy LTR-RTs are overrepresented according to a two-tail Fisher’s test (p-value = 9.81e-08) in tandem LTR-RT structures (88%, while these elements account for the 74% of the total LTR-RTs) and among gypsy elements the Tekay lineage seems also to be more prone to form tandem LTR-RT structures (66% of the LTR-RT tandems are related to Tekay elements although these elements account for 35% of the total LTR-RTs, an enriched found significative according to a one-tail Fisher’s test (p-value = 2.2e-16). These results are in line with what was found analyzing the genome of Nipponbare rice only (see Fig. 3). At the level of families, the Tekay families more represented among the tandem LTR-RTs are RIRE8A (24%), RETRO1 (11.5%), and RIRE3 (9.5%) (Additional Table 6).

### Using the cactus-minigraph pangenome for characterizing LTR-RT polymorphic structures

The pangenome approach described above allowed us to identify many tandem LTR-RT insertions present in rice and related species. However, this approach proved to be of limited use for the correct characterization of the different alleles these structures can produce. Indeed, the analysis of the TLI2 locus, which can be present in up to six different haplotypes, showed that this locus was not satisfactorily resolved in the pangenome. The different haplotypes were collapsed, as the different structural variants occur at the same position and have extensive sequence identity. Consequently, only two haplotypes were defined at this position, the LTR-RT tandem inserted within the MULE element present in the reference genome, and a deletion corresponding to the absence of insertion of both the MULE and the nested tandem LTR-RT structure (Additional Fig. 6). The accessions presenting other haplotypes were resolved as having one of these two, with the single LTR-RT insertion nested in the MULE (Hap 4, Fig. 2) being resolved as in the reference (which contains a tandem LTR-RT insertion, Hap 3), and the accessions presenting the insertion of the MULE alone (Hap 2), as deletions of the MULE and the nested LTR-RT structures (Hap 6).

**Fig. 5:**
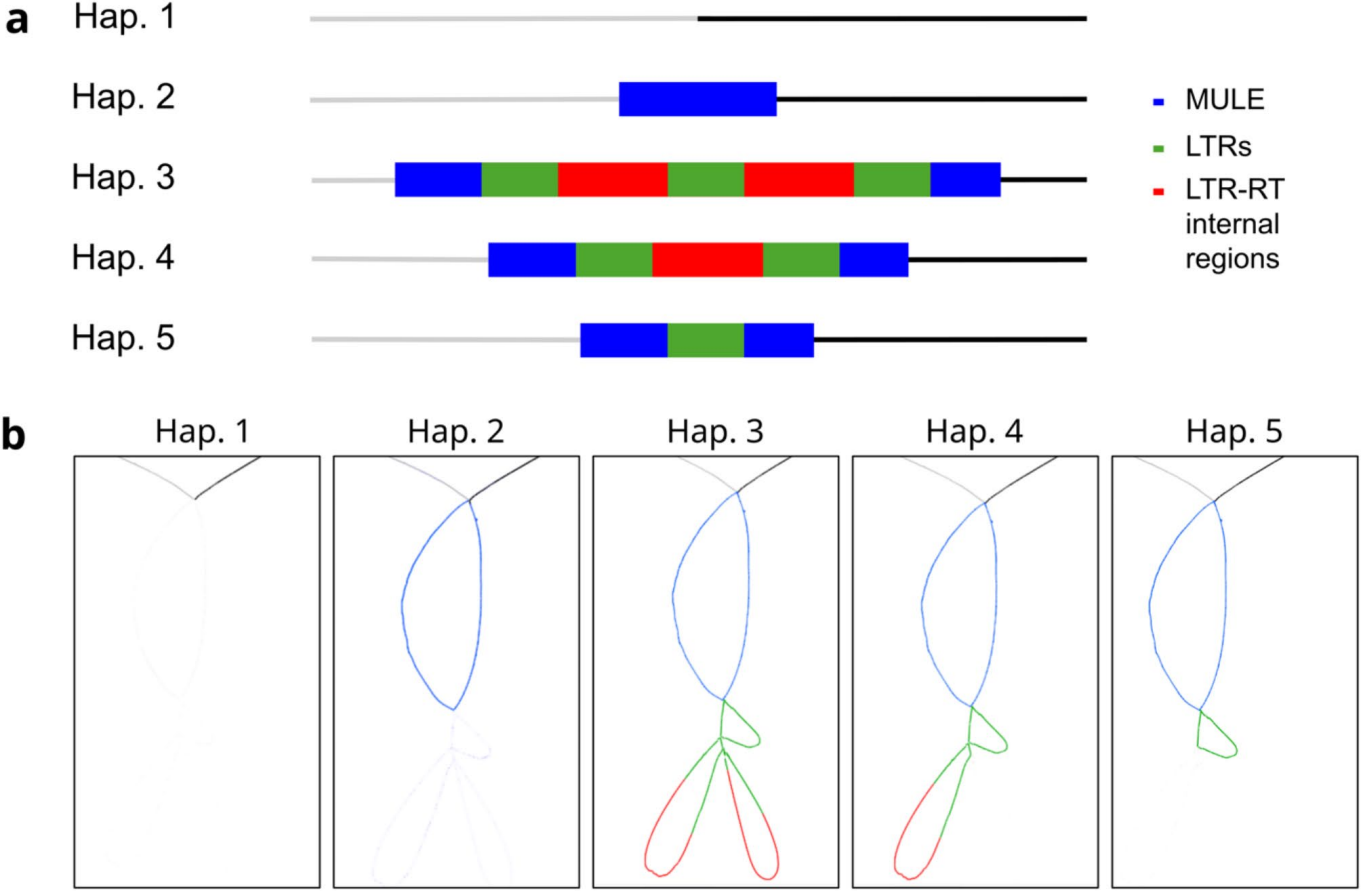
Bandage visualization of the TLI2 locus in the cactus-minigraph pangenome graph. **a**) Scheme of the different haplotypes identified in the graph. **b**) Visual representation of the different haplotypes using Bandage. The accessions used for the creation of the graph are described in Additional Table 2

This prompted us to use minigraph-cactus, which does not collapse duplications during the pangenome construction [33]. This pipeline allowed us to further resolve multiallelic, complex SVs. Figure 5 shows the minigraph-cactus pangenome version graph showing the complex allelic variants defined in Fig. 2, which could not be defined with the previous approach. As the Bandage visualization shows, all haplotypes previously defined are easily characterized using this approach except for the 29 bp deletion, as in the pipeline regions smaller than 50 bp were not considered SV (see Methods section).

An analysis of the 28 loci characterized here as containing tandem LTR-RT insertions using the cactus-minigraph pangenome showed that 61% of the tandem LTR-RT loci are fixed, while the rest are polymorphic, often giving rise to multiple haplotypes (up to 7 different haplotypes in a single locus), which highlights the high genomic diversity LTR-RT tandems can generate.

### LTR-RT tandems are common in plant genomes

**Fig. 6:**
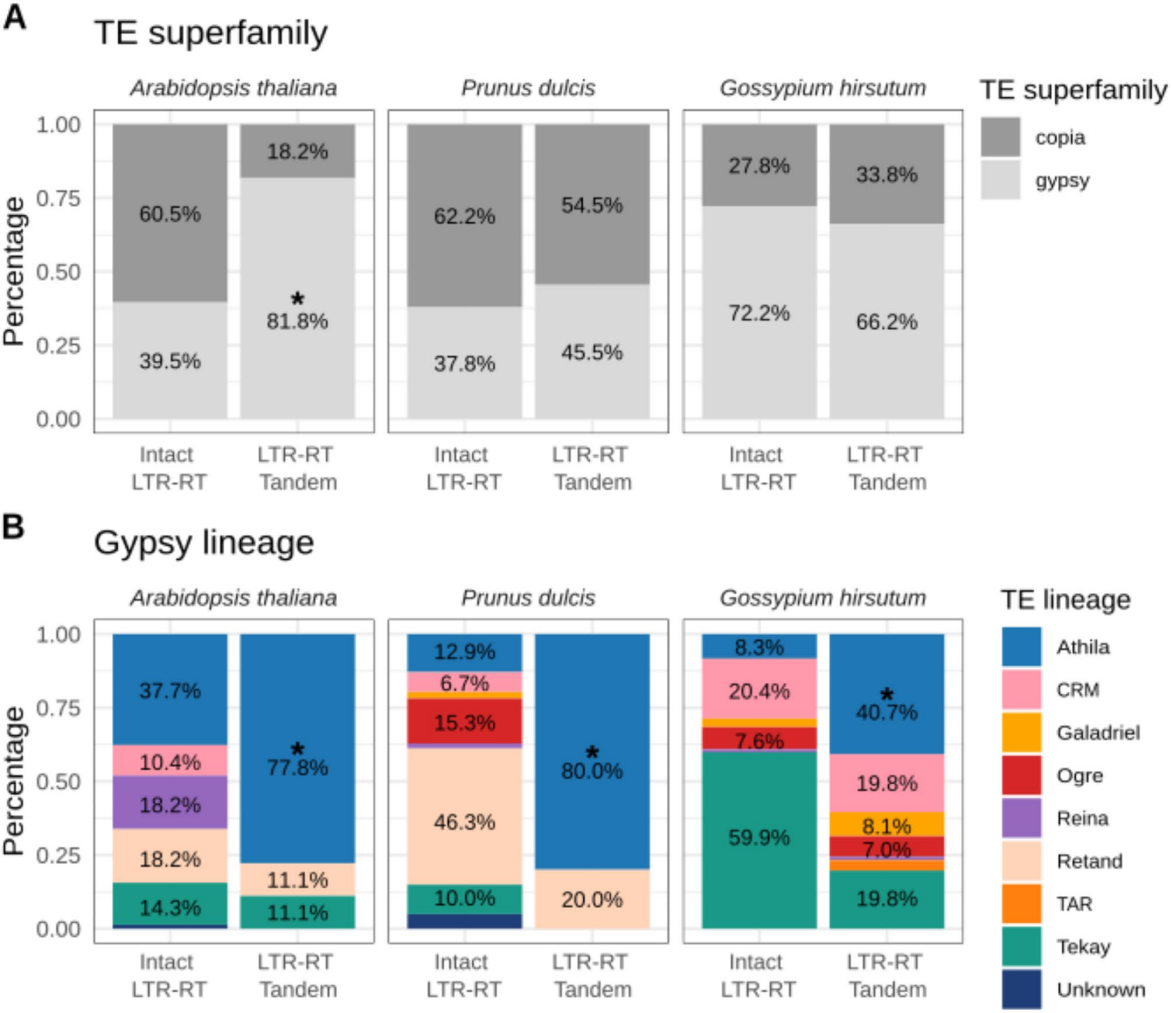
Intact LTR-RT and LTR-RT Tandems in *Arabidopsis thaliana*, *Prunus dulcis* and *Gossypium hirsutum*. genomes. **A**) Frequency of copia and gypsy elements identified as intact LTR-RT and LTR-RT tandem insertions in the three genomes. **B**) Frequency of the different gypsy lineages detected as intact LTR-RT and LTR-RT tandem insertions in the three genomes. The asterisk indicates statistical enrichment (*p* < 0.05) based on Fisher’s test

To evaluate how common the presence of tandem LTR-RT structures is in plant genomes we ran the IDENTAM pipeline on the assembled genomes of three other plant species including *Arabidopsis thaliana* (TAIR 10) [42], *Prunus dulcis* (almond) [43] and the upland cotton *Gossypium hirsutum* [44], which span a wide range of genome sizes, LTR-RT content and have different levels of ploidy. We found tandem LTR-RT structures in all of them, with a lower number in the genomes with a lower content of LTR-RTs (11 tandem LTR-RT structures in *A. thaliana* and *P. dulcis*) and higher in bigger genomes (e.g. 130 in cotton). With respect to the type of LTR-RT forming tandems, the analysis of these genomes shows that in in most of them gypsy LTR-RTs seem more prone to form tandem LTR-RT structures (Fig. 6). Indeed, tandem LTR-RTs are significantly enriched in gypsy elements in Arabidopsis (Fisher’s test p-value = 0.009041), as found in rice (Fig. 4), whereas in cotton and almond there is no significant enrichment for any of the two LTR-RT main superfamilies, gypsy and copia (Fig. 6).

Our analysis also shows that in most genomes there is a bias towards specific gypsy lineages to form LTR-RT Tandems (Fig. 6), but the specific lineage enriched depends on the genome analyzed. Athila elements are highly enriched in the LTR-RT Tandem group in *A. thaliana* (p-value = 0.02573), *P. dulcis* (p-value F0 = 0.001404) and cotton (p-value = 5.205e-16), whereas in rice the tandem LTR-RT structures are enriched in Tekay elements (p-value = 2.2e-16; Fig. 4).

The analysis of the rice pangenome suggested that these tandem LTR-RT structures are highly polymorphic within a species. Interestingly, the analysis of the phased genome of almond showed that among the 11 tandem LTR-RT structures identified in the F1 phase, one was not present in the F0 phase which, on the other hand, has one additional tandem LTR-RT structure, which stresses the high variability of these structures (data not shown).

## Discussion

TEs are widespread in eukaryote genomes and their mobilization and amplification is thought to have an important impact on genome structure and gene regulation. In plants, TEs are known to be a major driving force of genome evolution [45] and they can account for most of the genome space. In addition to autonomous elements, genomes contain defective elements, which become increasingly difficult to identify as they accumulate mutations [46], and are overrepresented as compared with active TEs. The repetitive nature of TEs has made their identification and study challenging, in particular on genome assemblies based mainly on short-read sequences. With long-read based reference genomes and pangenomes of different plant species becoming available, it is now possible to annotate and study TEs with much more detail. Here we show that LTR-RTs can form tandem arrays of alternating LTRs and LTR-TR internal regions, which are flanked by TSDs. Our results show that these structures are relatively abundant in rice and are also present in other genomes, of both monocot and dicot plants, with different genome sizes and ploidy levels. This suggests that tandem LTR-RT insertions are widespread in plant genomes, as they also seem to be in other higher eukaryotes such *as Drosophila* [24]. Our results suggest that gypsy elements tend to form more LTR-RT tandem structures than copia LTR-RTs, and some biases towards certain gypsy lineages seem also to exist, although different lineages seem prone to form these structures in different genomes. This general trend of gypsy elements could be the result of their average longer LTR size, that may more easily promote illegitimate recombination, or the frequent association of gypsy elements with heterochromatin and pericentromeric regions. However, we have not been able to detect any significant correlation of tandem LTR-RT formation and any of these features.

The pangenome-based analysis of the variability linked to these structures showed that they are highly dynamic, with more than 66% of the LTR-TR tandems found in Nipponbare being absent in at least one of the other *Oryza* genomes analyzed. Moreover, when present, LTR-RT tandems can generate many different haplotypes with a variable number of the tandemly repeated unit. This significantly expands the potential of LTR-RTs to generate genome variability within a species. Although these structures are more frequently located far from genes, some of them are found close to genes and therefore this genome variability may translate into phenotypic diversity. However, analyzing LTR-RT tandems at a population scale is complex and requires the use of completely assembled genomes and novel pangenome graph pipelines to properly their genetic variability.

Tandem LTR-RT structures like the ones described here have been found in the centromeres of two different species of kangaroos, and it has been proposed that they could arise by illegitimate recombination between the two different LTRs of the LTR of the element sitting in sister chromatids or homologous chromosomes, which could also give rise to solo-LTRs [20]. Indeed, the same mechanism was proposed to explain tandem arrays of TRIMs in different species, although it was also proposed that these structures could also result from the insertion of tandem structures produced during retrotransposition [18, 19]. Interestingly, it has recently been shown that the retrotransposition process involves the formation of circular LTR-RT DNA containing a single LTR that can be used for transcribing LTR-RT mRNA to initiate a new round of replication [47]. Under this scenario, the presence of a weak transcriptional terminator, as the one described for the tobacco Tnt1 LTR-RT [48] could allow the production of tandem LTR-RT transcripts, leading to the transposition of tandem LTR-RT structures. The analysis of the SNPs surrounding the insertion site in the 6 different haplotypes of the chromosome 2 locus, did not allow us to establish the complete sequence of events leading to diversity of structures present, and determine whether the tandem LTR-RT structure is the result of a complex insertion or of an illegitimate recombination event. At this point, both mechanisms seem possible and not necessarily mutually exclusive. More research will be needed to clarify this point.

## Conclusions

Tandem LTR-RT structures are widespread in plant genomes and can give rise to multiple haplotypes. The frequent and highly polymorphic nature of tandem LTR-RTs expands the potential of LTR-RTs to generate genome variability with potential phenotypic consequences.

## Electronic supplementary material

Below is the link to the electronic supplementary material.

Supplementary Material 1

Supplementary Material 2

Supplementary Material 3

Supplementary Material 4

Supplementary Material 5

Supplementary Material 6

Supplementary Material 7

## Data Availability

All data generated or analysed during this study are included in this published article [and its supplementary information files].
