# Supplementary material for: Tandem LTR-retrotransposon structures are common and highly polymorphic in plant genomes
Source: Mob DNA. 2025 Mar 12;16:10. doi: 10.1186/s13100-025-00347-y (PMC11899658; doi:10.1186/s13100-025-00347-y)
Supplement: Supplementary file 1 — Supplementary Material 1 [file 13100_2025_347_MOESM1_ESM.docx]

**Additional Figures and Tables**


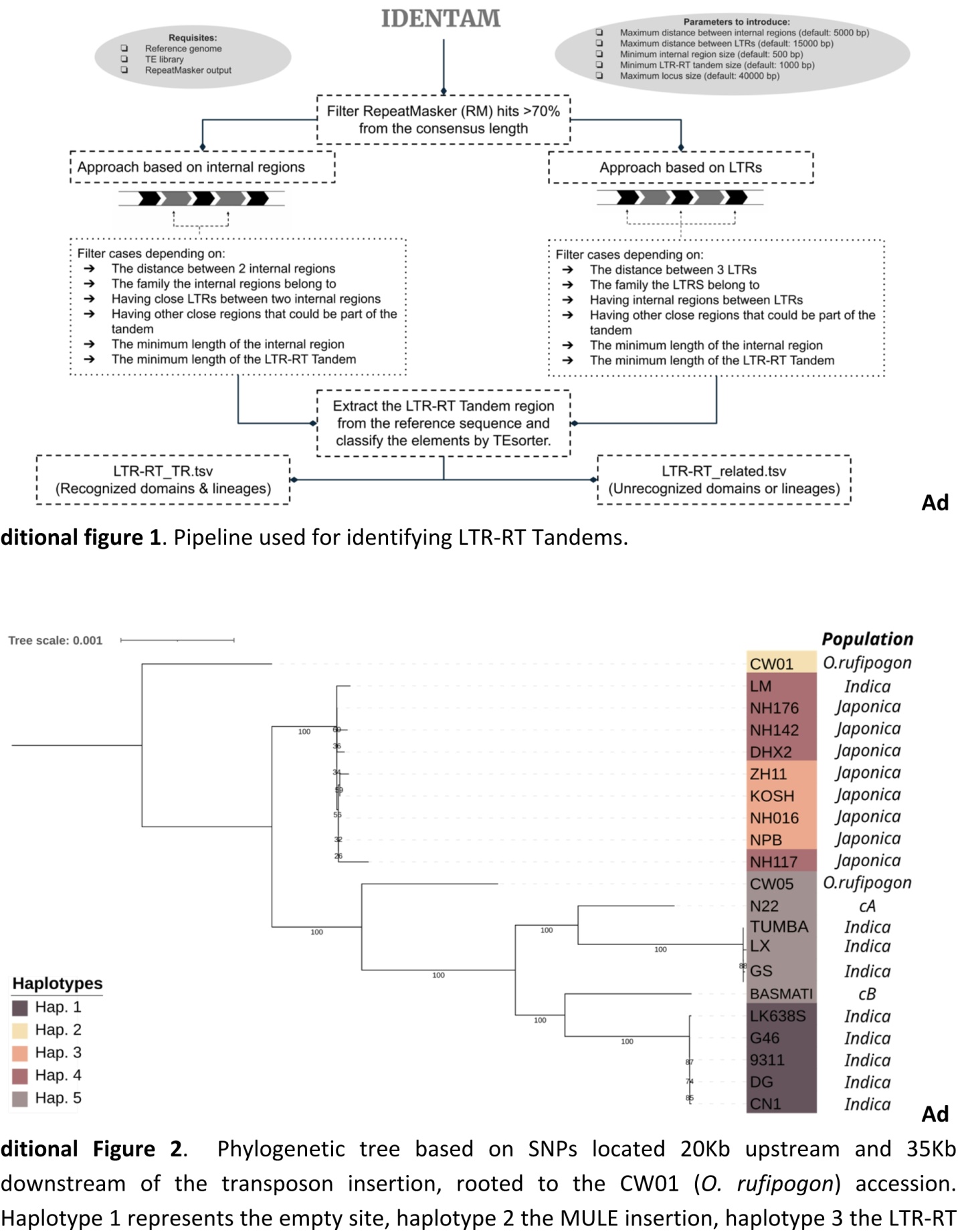


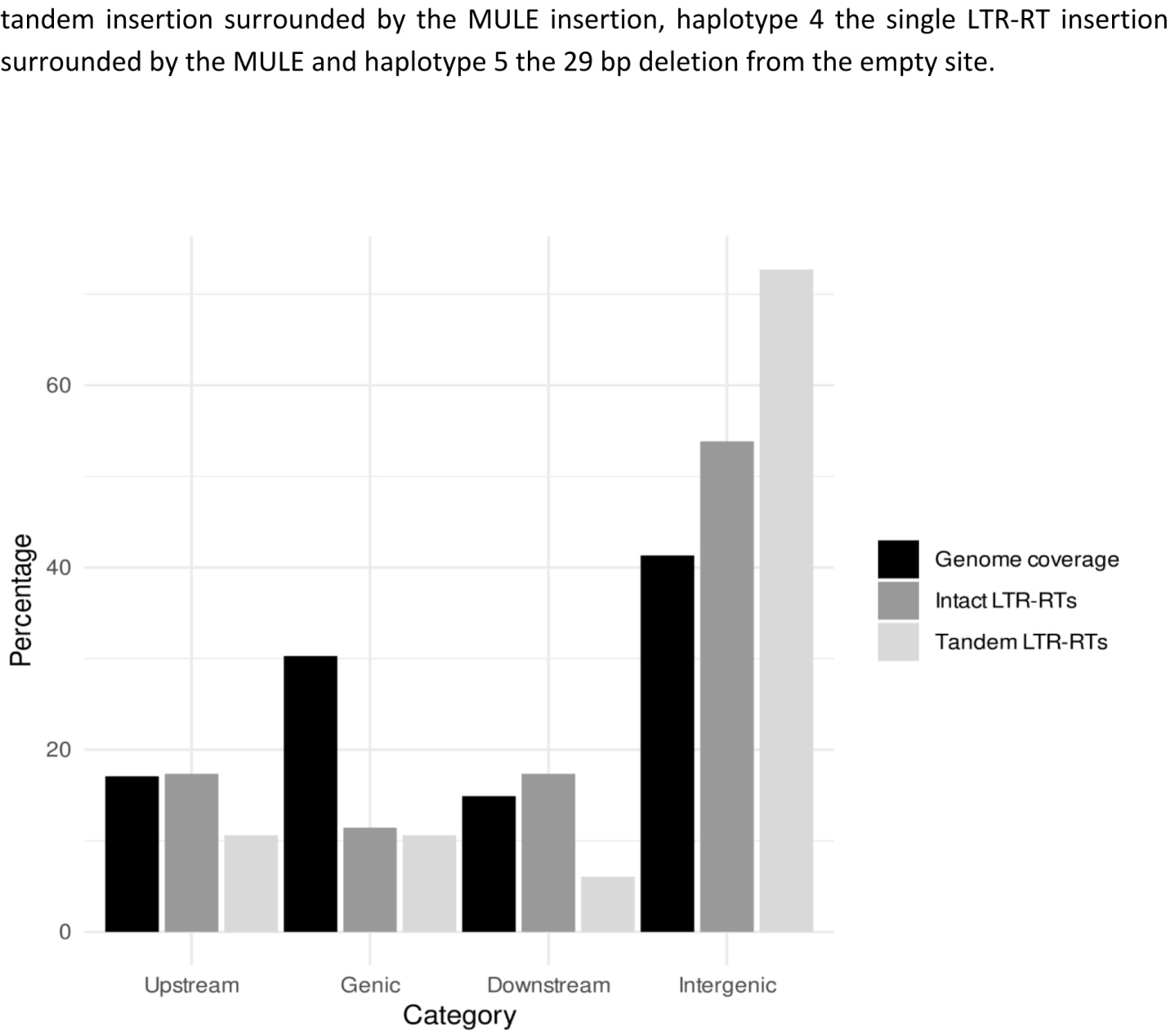


**Additional Fig. 3.** Distribution of LTR-RT tandems and LTR-RT intact elements with respect to genes in Nipponbare rice


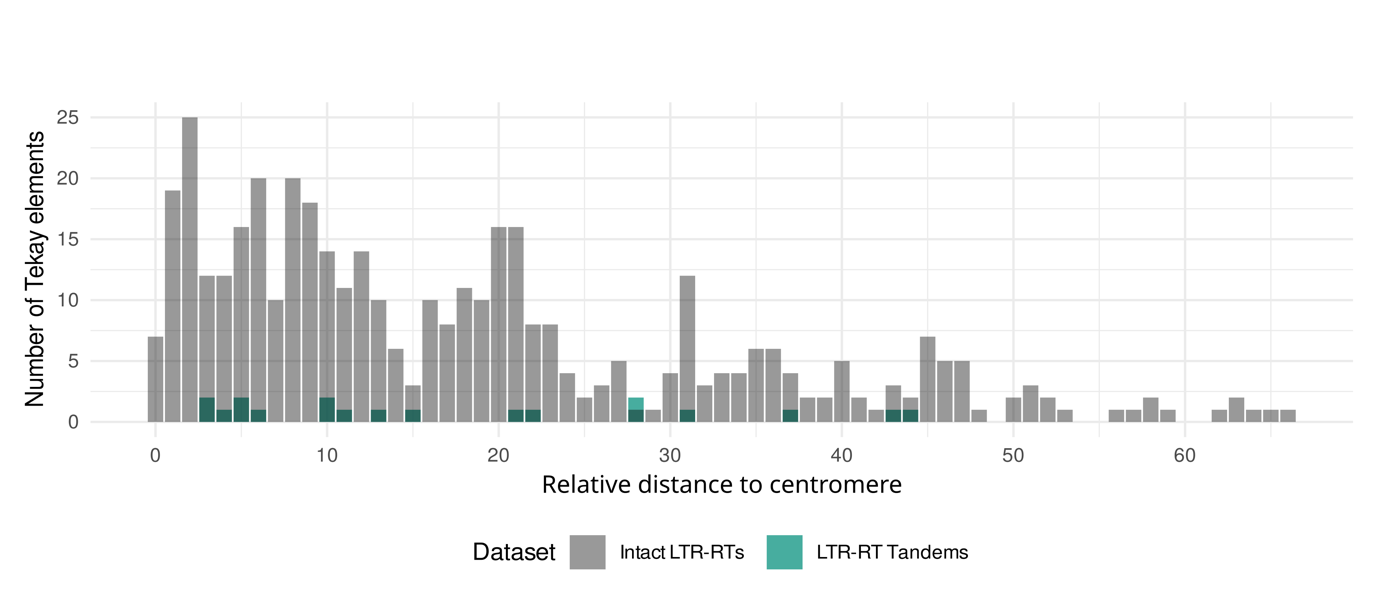
**Additional Fig. 4**. Distribution of Nipponbare rice Tekay intact and tandem LTR-RT relative to the centromere. The graph shows the number of Tekay transposons plotted against their relative distance to the centromere.


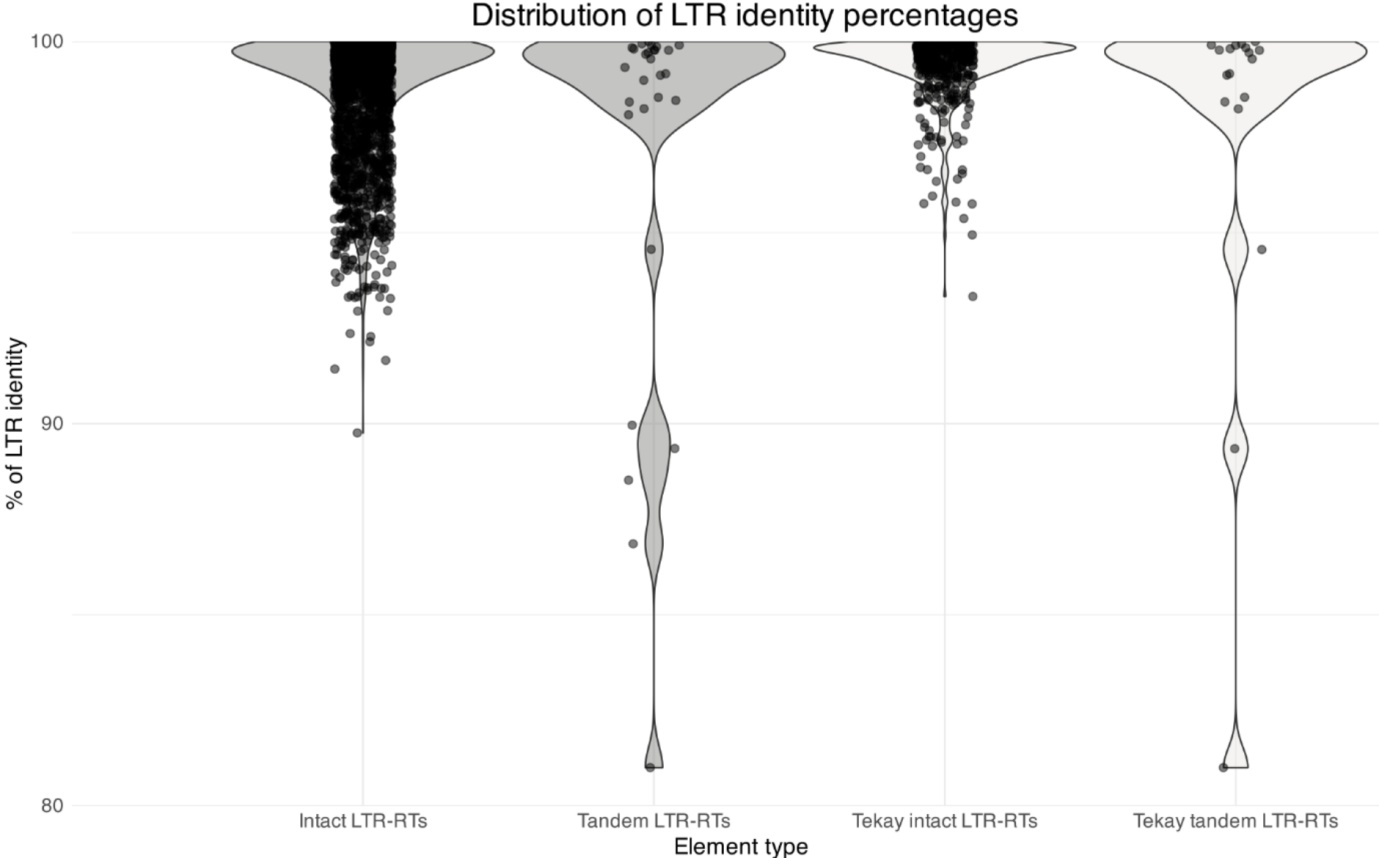


**Additional Fig. 5**. Distribution of intraelement LTR identity of intact LTR-RT and tandem LTR-RT


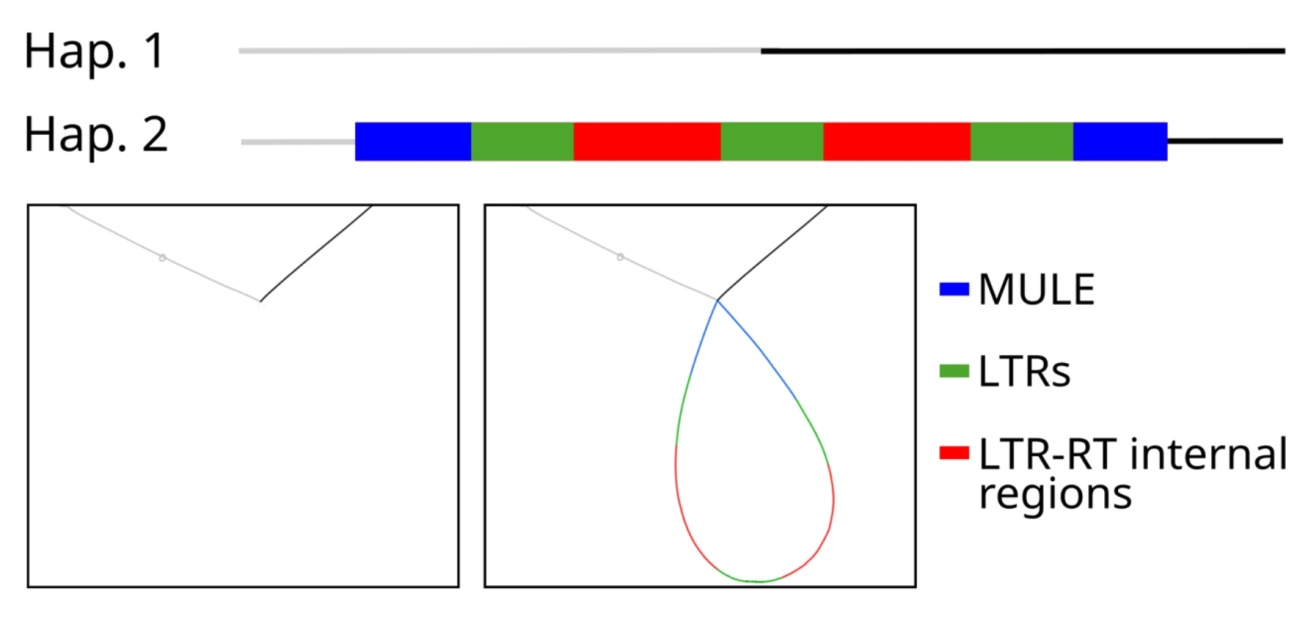


**Additional Fig. 6**. Bandage visualization of the minimap2-SVIM pangenome in Chr02:359587–381449 and scheme of the different haplotypes identified in the graph. The accessions used for the creation of the graph are described in Additional Table 2.

Additional Table 1. Accessions used for pangenome construction using minimap2 + SVIM-asm

Additional Table 2. Accessions used for pangenome construction using cactus-minigraph

Additional Table 3. Information on the structure of the TLC2 locus in the genomes of different rice accessions and related species.

Additional Table 4. Description of the lineages and families of the 66 tandem LTR-RT structures from Nipponbare

Additional Table 5. Selected LTR-RT tandems present in the Nipponbare genome.

Additional Table 6. Description of the lineages and families of the 200 tandem LTR-RT structures from the rice pangenome.
